# The impact of EEG biofeedback training on the athletes’ motivation and bench press performance

**DOI:** 10.5114/biolsport.2024.127065

**Published:** 2024-01-02

**Authors:** Magdalena Prończuk, Tomasz Chamera, Jarosław Markowski, Jan Pilch, Wojciech Smólka, Adam Zajac, Adam Maszczyk

**Affiliations:** 1 Institute of Sport Sciences, The Jerzy Kukuczka Academy of Physical Education, Katowice, Poland; 2 Faculty of Physical Culture, Gdańsk University of Physical Education and Sport, Poland; 3 Department of Laryngology, Faculty of Medical Sciences, Medical University of Silesia in Katowice, Poland; 4 Department of Physiological and Medical Sciences, The Jerzy Kukuczka Academy of Physical Education, Katowice, Poland

**Keywords:** EEG biofeedback, Bench press, Motivation

## Abstract

The objective of this paper was to determine the impact of EEG-biofeedback training on the motivation and efficiency of powerlifters during the bench press exercise in relation to the external load and the level of training. The study included 18 trained powerlifters who were divided into the intermediate (IG) and the advanced (AG) groups. EEG-biofeedback training was conducted every three days, lasting 27 minutes each time (5 × 3-minute intervals with recovery periods – lying on a bench – between them 4 × 3 minutes), and ended with a final EEG measurement in the second cycle of research. The repeated measures ANOVA showed intra-group differences due to external loading for the FAI (Frontal Alpha Asymmetry) obtained in the EEG both before and after biofeedback training. In AG group analysis revealed significant differences between 65%1RM and 35%1RM. In the IG group between 35%1RM and 50, 65 and 80%1RM. One of the major variables influencing the efficiency of strength training, including bench press workouts, is the level of training. The more successfully an athlete uses motivation when exercising, the better their training, which translates into greater results and a lower chance of injury.

## INTRODUCTION

Athletic strength is determined by a complex interaction of various factors, including motivation, EEG-biofeedback training, and external load. The way in which these factors affect each other can have significant implications for an athlete’s ability to perform at their best. In particular, the impact of differences in motivation on performance can be substantial, especially when it comes to high-intensity activities such as weightlifting. The bench press exercise is a staple in many strength and conditioning programs, and it requires both physical and mental effort to perform correctly.

The link between perception and thinking expresses motivation as a certain mental state of tension. In psychological terms, a mental process, is the foundation of involvement in activities, translating directly into the quality and effect of action, which, in the case of sports, can influence the athlete’s performance [1]. Concentration plays a crucial role in strength sports, which are characterized by a tendency to continually enhance the level of technique of the performed task. Attention is a neuropsychological process consisting of a specific concentration of cognitive functions on a particular task or the precise awareness of active inputs [2]. Athletes at the professional level experience less satisfaction from the training phases, which may be a reflection of their understanding of the fundamental goal of sports practice. Sufficiently strong motivation focused on a specific objective in an era of regular, frequently repetitive training, is a crucial psychological factor impacting elite training [3]. The brain’s bioelectrical activity may reflect mental states, including, in the case of sports, the athlete’s capacity to perform a motor task. The bioelectrical activity of the brain is dynamic and can be influenced by both external and internal factors, such as the athlete’s mental state. Electroencephalography is able to monitor this process (EEG). Changes in brain electrical activity are seen in real-time, and distinct frequency ranges in specific parts of the cortex. They are associated with a variety of emotional states, including motivation [4, 5]. The motivational factor is very significant in terms of performance, especially with progressive loads in powerlifters. Bench press (BP) is a complicated multi-joint exercise that activates several upper-body muscle groups, allowing to lift large external loads that demand a high level of neuromuscular activation. One of the most popular powerlifting events is the bench press because it is a separate competition in which the world championships are held. The results produced by strongmen in this competition are mostly the product of developed motor abilities, technique, and dedication (mental attitude). Due to the strength-building potential of the bench press and the popularity of bench press competitions, it is frequently utilized for training, testing, and research [6]. Bench press kinematics [7], the effectiveness of various chest exercises [8], the impact of an unstable surface on upper body muscle activation [9], effects of fatigue [10], and motivational analysis of BP exercises with maximum and submaximal loads have been the subject of previous research [11, 12, 13].

Training athletes’ motivation can be associated with various aspects of brain functioning, including those related to EEG (electroencephalography) and FAI (functional asymmetry of the brain). FAI refers to differences in electrical activity between the two hemispheres of the brain. A typical phenomenon measured in FAI is the asymmetry of activity between the left and right hemispheres. Research in the field of sports psychology suggests that athletes’ motivation may be related to the asymmetry of brain activity, especially in areas related to emotions and reward processing. Some studies suggest that greater activity in the left hemisphere of the brain may be associated with higher motivation and positive emotions, while greater activity in the right hemisphere may be associated with negative emotions and lower motivation [3,4,5].

In the context of training athletes’ motivation, monitoring FAI using EEG can be useful for understanding which training strategies are most effective for a specific athlete. If an athlete shows lower motivation and greater activity in the right hemisphere of the brain, coaches can focus on strategies aimed at balancing this brain activity through training techniques that may promote greater activity in the left hemisphere of the brain, such as positive reinforcement, visualization of success, or relaxation techniques [2,3]. In this way, tracking FAI through EEG can be a diagnostic tool in sports psychology, helping to tailor training strategies to the individual needs and brain characteristics of the athlete, which can lead to improvements in their motivation and sports achievements [2,3,4,5].

Resuming, athletic performance is determined by a complex interaction of various factors, including motivation, training, and external load. The way in which these factors affect each other can have significant implications for an athlete’s ability to perform at their best. In particular, the impact of differences in motivation on performance can be substantial, especially when it comes to high-intensity activities such as weightlifting. The bench press exercise is a staple in many strength and conditioning programs, and it requires both physical and mental effort to perform correctly.

Therefore, the aim of this study was to determine the impact of EEG-biofeedback training on the motivation and efficiency of powerlifters during bench press exercises in relation to the external load and training level.

## MATERIALS AND METHODS

### Participants

The study included 20 trained powerlifters with a minimum of 4 years of training experience (± 0.5 years) and 20 powerlifters with at least 7 years of training experience (± 0.5 years). Age 24 ± 0.5 years and right-handedness were additional criteria for inclusion in the research groups (scores above 35 points on a scale of 39 points) [14]. Russkam expresses 29 moods and emotions with three levels of intensity [15]. Based on the results of the Russkams set, we removed four athletes (two from each group) from further testing. We made this decision whenever the participant marked feeling at least one of the negative emotions at the highest level. Two study groups were formed as a consequence of the random selection of every second competitor from each list for the appropriate tests: the intermediate group (IG n = 9) and the advanced group (AG n = 9) (Table 1). The participants were informed orally and in writing about the experimental methodology, and the potential of withdrawal at any time, and provided written consent to take part in the study. EEG was used to conduct the measurements in the Laboratory of Psychomotor Fitness and the Laboratory of Muscular Strength and Power at the Academy of Physical Education in Katowice. The University Bioethics Committee for Research (7/2016 for NRSA 4 040 54) approved the research.

**TABLE 1:** Characteristics of the study groups (n = 18)

Group	Advanced group		Intermediate group		p

Variable	±	SD	±	SD	
BM (kg)	82	2.512	81	1.821	0.781
BH (cm)	181	3	183	4	0.801
BMI (index)	24	1.721	25	1.688	0.832
PBF (%)	7	1.621	8	1.501	0.845
SMM (kg)	45	2.114	42	2.601	0.796

BM-Body mass; BH-Body height; BMI – Body Mass Index; PBF-Body fat percentage; SMM-Skeletal muscle mass.

### Research procedures

EEG measurements were conducted after a 72-hour break from any weightlifting exercises. The studies were conducted in two cycles. Individual measurement cycles were conducted over a 12-day period (each), with 3 athletes per day. The interval between cycles was 8 weeks. The first cycle of research included an initial EEG measurement based on the amount of external load. Next, biofeedback training was conducted for each athlete individually for a period of 8 weeks (15 biofeedback training sessions). We trained on Fp1, Fp2 and Cz points, promoting beta1 and SMR rhythms respectively. For each competitor, we also checked the peak alpha frequency (PAF). The frequency ranges did not require correction.

Motivational training was conducted every three days, lasting 27 minutes each time (5 × 3-minute intervals with recovery periods – lying on a bench – between them 4 × 3 minutes), and ended with a final EEG measurement in the second cycle of research.

### EEG Measurement

The EEG recordings were conducted according to the procedures of the International Federation of Clinical Neurophysiology and the American EEG Society. The EEG was recorded using AG/AgCl scalp electrodes, placed according to the international 10–20 system [16]. The EEG recording was made on a 24-channel Deused Truscan system. The sampling frequency was 1024/s. Electrode impedance was maintained below 5 kilohms. A 50 Hz mains filter and a high and low-pass filter (respectively 1 and 40 Hz) were used. The grounding electrode was placed on the ear. An airplane cushion pillow was placed on the bench to reduce muscle artifacts on the neck. The measurement started with a 2-minute EEG recording at rest. First, the recording with closed eyes was checked due to epileptiform electroencephalographic patterns or other abnormalities. The rest of the recording and the entire analysis were performed with open eyes.

Fifteen seconds before each bench press, attempt the subject’s task was to focus and motivate on the task. The rest intervals between sets were 5 minutes. 15-s segments of the recording before the attempt from electrodes F3 and F4 were analyzed and manually divided into 1-s parts epochs. Those showing artifacts from additional co-occurring muscle activity were removed. The average result was subjected to the Fast Fourier Transform. Spectral power (μV2) at the alpha range (8–13 Hz) was exported. Finally, the alpha power of EEG electrodes F3 and F4 was log-transformed and the asymmetry score of frontal alpha was calculated by subtracting the value at F3 from the value at F4. Therefore, the Frontal Alpha Asymmetry Index (FAI) was calculated for each concentration phase before lifting the barbell according to the formula proposed by Coan and Allen [17].

Each EEG recording was assessed by a neurologist certified by the Polish Society of Clinical Neurophysiology, who was blinded for the subjects.

### The Bench press procedures

The measurements were taken in 5 sessions with a 3-minute rest interval between them to avoid potential effects of fatigue. The sessions consisted of performing one repetition of a flat bench press barbell with weights ranging from 35% of one repetition maximum (1RM) to 100% 1RM. A standard warm-up protocol was applied for each session, including a general warm-up (5 minutes) on a handcranked cycle ergometer (heart rate at around 130 beats per minute) and several strength exercises without external load that engaged the upper and lower body. The 1RM value was determined according to the Tilliar and Saeterbakken [10] protocol. The protocol included a “free” barbell press on a horizontal bench. When approaching the barbell press, the participants lay on their backs with EEG connected, their heads resting on an inflatable pillow, their trunk supported on the bench, their knees bent at a right angle at the knee joint, and their feet resting on the floor. The barbell grip width, similar to the static test, was 81 cm between the index fingers and was the maximum allowed by the rules of the International Powerlifting Federation. One person (an experienced coach) controlled and secured the participants during the press. The participants were instructed to lower the barbell in a controlled manner until it touched the chest and then, without stopping, to push it upward until full elbow joint extension. The exercise protocol consisted of a total of 5 sessions that allowed the determination of the variables: S35 – 1 repetition with 35% 1RM load; S50 – with 50% 1RM load; S65 – 1 repetition with 65% 1RM load, S80 – 1 repetition with 80% 1RM load, and S100 – 1 repetition with 100% 1RM load. The rest period between sessions were 3 minutes, and the participants lay on the bench.

### Statistical Analysis

Descriptive statistics, such as the arithmetic mean, standard deviation, and coefficient of variation, were used to evaluate the level of analyzed variables. The normality of the variables’ distribution was checked using the Shapiro-Wilk test. Levene’s test of homogeneity of variances was applied to verify the homogeneity of variables and determine the statistical tools. The results of the tests clearly indicated that the variables had a normal or near-normal distribution (p > 0.05).

The homogeneity of variances was examined before and after the training sessions using Levene’s test and it showed no similarities, i.e., homogeneity for all variables. The values of the variables in both groups after the training sessions in the Levene test were also homogeneous.

Repeated measures ANOVA was applied to answer questions regarding testing hypotheses about the absence of differences between the values of the individual variables describing the intergroup and intragroup relationships.

The F statistic and level of significance were presented. A significance level of p < 0.05 was accepted. Tukey’s post hoc tests for equal sample sizes (N) were performed in case of significant differences.

All calculations were performed using the Statistica 15.0 analytical program (Statsoft) and the Excel package (Microsoft Office 13).

## RESULTS

Repeated measures ANOVA showed no intra-group differences due to external loading for RH (right hemisphere) and LH (left hemisphere) variables obtained in EEG both before and after the biofeedback training.

**FIG. 1:**
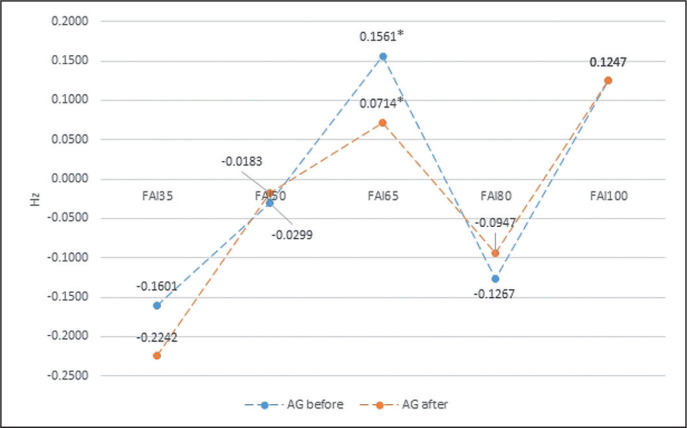
The intra-group differences in FAI (Frontal Alpha Asymmetry) before biofeedback training, in relation to an external load for AG group. * statistically significant differences.

**FIG. 2:**
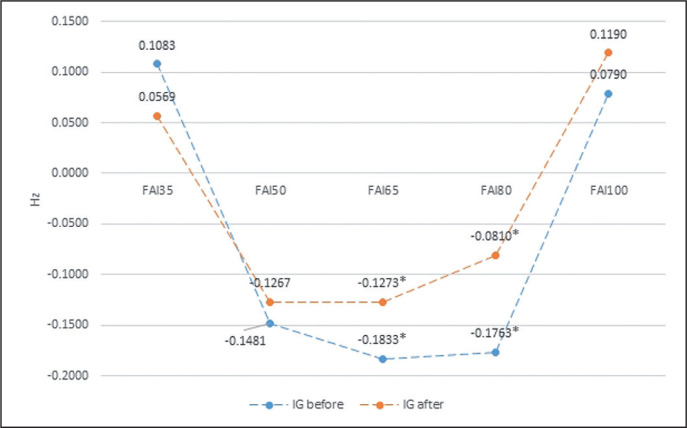
The intra-group differences in FAI (Frontal Alpha Asymmetry) after biofeedback training, in relation to an external load for IG group. * statistically significant differences.

The same analysis shows intra-group differences due to external loading for the FAI (Frontal Alpha Asymmetry) obtained in the EEG both before and after the biofeedback training. In the AG group, the analysis revealed significant differences between 65%1RM and 35%1RM, while in the IG group significant differences between 35%1RM and 50, 65, and 80%1RM (Tables 3 and 4, Figures 1 and 2).

**FIG. 3:**
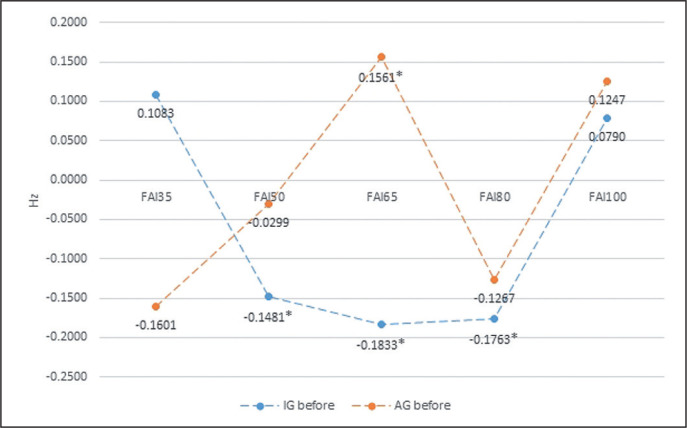
The intergroup differences in FAI (Frontal Alpha Asymmetry) before biofeedback training, in relation to an external load. * statistically significant differences.

**FIG. 4:**
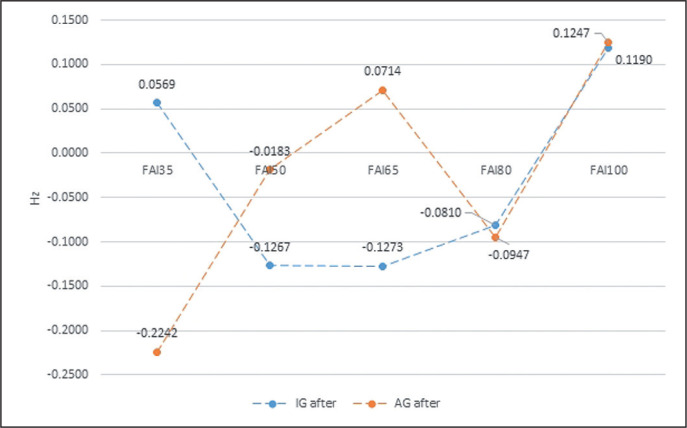
The intergroup differences in FAI (Frontal Alpha Asymmetry) before biofeedback training, in relation to an external load. * statistically significant differences.

At the same time, the repeated measures ANOVA showed significant intergroup differences due to external load for the RH after biofeedback training and for the FAI both before and after biofeedback training (Tables 2 to 4, Figures 3 and 4).

**TABLE 2:** The result of the intergroup analysis of variance with repeated measures for the RH (right hemisphere) after biofeedback training, in terms of external load

Group	Advanced group						Intermediate group				

	External load	35%1RM	50%1RM	65%1RM	80%1RM	100%1RM	35%1RM	50%1RM	65%1RM	80%1RM	100%1RM
AG	35%1RM		0.510	0.550	0.989	0.120	0.937	0.989	0.989	0.989	0.855
AG	50%1RM	0.510		0.989	0.983	0.999	0.999	0.165	0.821	0.796	0.989
AG	65%1RM	0.550	1.000		0.989	0.998	0.999	0.186	0.850	0.827	0.989
AG	80%1RM	0.989	0.983	0.989		0.683	1.000	0.809	0.989	0.989	0.989
AG	100%1RM	0.120	0.999	0.998	0.683		0.866	0.022	0.327	0.301	0.943

IG	35%1RM	0.937	0.999	0.999	0.989	0.866		0.606	0.997	0.996	0.989
IG	50%1RM	0.989	0.165	0.186	0.809	0.022	0.606		0.981	0.986	0.456
IG	65%1RM	0.989	0.821	0.850	0.989	0.327	0.997	0.981		0.989	0.984
IG	80%1RM	0.989	0.796	0.827	0.989	0.301	0.996	0.986	0.989		0.979
IG	100%1RM	0.855	1.000	0.989	0.989	0.943	1.000	0.456	0.984	0.979	

IG – intermediate group, AG advanced group.

**TABLE 3:** The result of the intra and intergroup analysis of variance with repeated measures for the FAI (Frontal Alpha Asymmetry) before biofeedback training, in relation to an external load

Group	Advanced group						Intermediate group				

	External load	35%1RM	50%1RM	65%1RM	80%1RM	100%1RM	35%1RM	50%1RM	65%1RM	80%1RM	100%1RM
AG	35%1RM		0.915	0.028	0.989	0.071	0.111	0.989	0.989	0.989	0.226
AG	50%1RM	0.915		0.575	0.987	0.795	0.882	0.952	0.802	0.842	0.971
AG	65%1RM	0.028	0.575		0.075	0.989	0.989	0.040	0.013	0.016	0.998
AG	80%1RM	0.989	0.987	0.075		0.170	0.247	0.989	0.989	0.989	0.430
AG	100%1RM	0.071	0.795	0.989	0.170		1.000	0.099	0.036	0.044	0.989

IG	35%1RM	0.111	0.882	0.989	0.247	0.989		0.015	0.035	0.049	0.989
IG	50%1RM	0.989	0.952	0.040	0.989	0.099	0.015		0.989	0.989	0.290
IG	65%1RM	0.989	0.802	0.013	0.989	0.036	0.035	0.989		0.989	0.130
IG	80%1RM	0.989	0.842	0.016	0.989	0.044	0.039	0.989	0.989		0.155
IG	100%1RM	0.226	0.971	0.998	0.430	0.989	0.989	0.290	0.130	0.155	

IG – intermediate group, AG advanced group.

**TABLE 4:** The result of the intra and intergroup analysis of variance with repeated measures for the FAI (Frontal Alpha Asymmetry) after biofeedback training, in relation to an external load

Group	Advanced group						Intermediate group				

	External load	35%1RM	50%1RM	65%1RM	80%1RM	100%1RM	35%1RM	50%1RM	65%1RM	80%1RM	100%1RM
AG	35%1RM		0.272	0.029	0.819	0.003	0.034	0.965	0.966	0.751	0.003
AG	50%1RM	0.272		0.995	0.997	0.809	0.997	0.953	0.951	0.999	0.843
AG	65%1RM	0.029	0.995		0.731	0.999	0.989	0.450	0.446	0.801	0.989
AG	80%1RM	0.819	0.997	0.731		0.262	0.767	0.989	0.989	0.989	0.297
AG	100%1RM	0.003	0.809	0.999	0.262		0.998	0.106	0.104	0.326	0.989

IG	35%1RM	0.034	0.997	0.989	0.767	0.998		0.025	0.040	0.042	0.999
IG	50%1RM	0.965	0.953	0.450	0.989	0.106	0.025		0.989	0.989	0.124
IG	65%1RM	0.966	0.951	0.446	0.989	0.104	0.040	0.989		0.989	0.122
IG	80%1RM	0.751	0.999	0.801	0.989	0.326	0.042	0.989	0.989		0.366
IG	100%1RM	0.003	0.843	0.989	0.297	0.989	0.999	0.124	0.122	0.366	

IG – intermediate group, AG advanced group.

Table 5 shows the results of the bench press exercise before and after 8 weeks of EEG-biofeedback training for both groups.

**TABLE 5:** Results of the bench press exercise before and after biofeedback training in AG and IG groups with statistical significant intra-group differences

	Advanced group			Intermediate group		

	Results before (kg)	Results after (kg)	p for AG	Results before (kg)	Results after (kg)	p for IG
35%1RM	82	86	0.456	75	84	0.175
50%1RM	118	123	0.501	108	120	0.089
65%1RM	153	159	0.389	140	156	0.048^*^
80%1RM	188	196	0.278	172	192	0.035^*^
100%1RM	235	245	0.154	215	240	0.028^*^

* statistically significant differences; IG – intermediate group, AG advanced group.

## DISCUSSION

Athletes use a variety of methods and tools to improve their performance and skills. One such innovation includes EEG biofeedback, a training method that statistically monitors brain activity to improve the efficacy of athletic training and, consequently, athletic performance [18, 19, 20, 21, 22]. The studies presented in this paper examine the effectiveness of bench press training using EEG biofeed-back, as well as the impact of athletes’ motivation, external load, and training intensity (15 biofeedback sessions – 8 weeks).

One of the most important elements affecting the effectiveness of a training program is the athlete’s motivation. Highly motivated athletes are more committed to their training and perform better [23, 24]. It has been proven that motivated athletes are more likely to perform exercises correctly, which increases their [25, 26].

The research presented in this paper shows a statistically significant difference of the increasing external load in terms of the strength of positive motivation, especially in the case of loads of 50% 1RM, 60% 1RM, and 80% 1RM. Similar results were obtained by other researchers [27, 28]. This applies not only to athletes who practice strength sports. The dependencies of the external load in terms of motivation and its use to improve the effectiveness of training have been and are used in team sports games and athletic competitions [12, 13, 18]. The size of the external load is an important factor affecting the effectiveness of training. According to scientific studies, muscle mass, and strength increase with progressive external loads [29]. However, athletes’ motivation can be undermined by overuse, and unwanted injuries can occur. As a result, it is essential to select the external load properly during training based on the athlete’s fitness level [30, 31].

The level of training also has a parallel effect on the effectiveness of training. Well-trained athletes perform better and are less likely to be injured. Scientific studies have shown that the level of training affects the effectiveness of training. An athlete’s ability to motivate themselves during exercise increases with the level of training, which translates into improved exercise performance.

The research presented in the paper shows that even small external loads significantly change the athlete’s motivation, especially in people with less training experience. The motivation of athletes, especially those with less training experience, decreases with increasing external load. The procedure is different for experienced athletes. At 80% 1RM and 100% 1RM, motivation increases most significantly (determined by EEG). Although the standard deviation was not excessive, the biofeedback training aimed to improve the FAI and thus increase motivation. However, in this study, the effect of this training was particularly visible in the group of IG players. Particularly significant changes in the improvement of bench press efficiency after biofeedback training were obtained for external loads of 65–100% 1RM. In the AG group, changes for the better were also noted, but they were not statistically significant. Similar results have been demonstrated by Standage and Ryan [32] and Xu [33]. This is because players with extensive experience are not as susceptible to biofeedback training as less experienced players. Probably, a much stronger training stimulus must be used in the training of advanced players [32, 33].

However, it is important to note that the amount of time an athlete spends exercising is not the only factor that determines their level of preparation. While consistent and adequate training is crucial for improving performance, it is also important to consider other factors such as nutrition, sleep, and recovery. People who are just starting strength training may have reduced exercise efficiency because they have not yet developed the necessary skills and muscle adaptations. On the other hand, advanced athletes who have been training for years may experience a plateau in their performance due to over-training or inadequate recovery [34].

Moreover, the level of training is not only influenced by the amount of time an athlete spends training, but also by the quality and specificity of the training. Athletes who set specific and challenging goals for themselves, and who vary their training to target different aspects of performance, are more likely to improve their overall level of fitness and performance. Additionally, athletes who are under pressure to perform at their best may invest more time and energy into their training, but it is important to balance this with adequate rest and recovery to avoid burnout and injury. Overall, a combination of consistent, high-quality training, proper nutrition, recovery, and goal-setting is essential for optimizing athletic performance [34, 35].

There is one more aspect that is very important and affects the overall success of sports training and biofeedback, namely the motivational climate [36]. In addition to an individual’s goal orientations, the particular environment or motivational climate created by the teacher, coach, peers, or parents can induce a state of task or ego involvement in sports and exercise situations. Gillet et al. [37] contended that the perceived motivational climate influences an individual’s thoughts, feelings, and achievement behaviors. Consistent with task and ego goal orientations, two climates have been found to be dominate in sports and educational environments: a performance (ego) climate and a mastery (task) climate. Research into perceptions of the motivational climate in sports and physical education (e.g. Lazarus [38]) has demonstrated that perceptions of a mastery climate are related to a task goal orientation, intrinsic motivation, a preference for challenging tasks, and beliefs that success is due to effort. Furthermore, a positive attitude, high satisfaction, low boredom and anxiety, high self-rated improvement, continued involvement, and self-determined reasons for participation have also been associated with perceptions of a mastery climate. The topic of the motivational climate was not the subject of this study, however, in the authors’ opinion, such an important aspect had to be indicated.

Over the past 40 years, research on motivation and self-perception, or self-confidence, has produced some significant findings [39]. The first step is to use motivation to push through loads that are heavier than the athlete can handle. There may be several scenarios, but only one stands out. Low-ego athletes might not use biofeedback training in a sensible manner. This is especially true when athletes feel inadequate and fear failure. This is likely to result in maladaptive behaviors that affect training efficacy. The research findings unmistakably show that under such conditions, motivation wanes, commitment to tasks wanes, perseverance wanes, efficiency wanes, satisfaction and joy wanes, relationships with teammates and coaches wane, burnout is more likely, and athletes feel worse about themselves and their accomplishments [39]. This is true for athletes who lack motivation or whose motivation is used inappropriately. So, it’s critical to gauge each situation’s level of motivation at first [39].

EEG-biofeedback training can even be more beneficial. Positively motivated athletes with high self-esteem who have a good sense of their own competence are able to greatly boost the effectiveness of training [40] self-ego’s motivational function in athletes causes them to engage in persistent performance-related actions. But even then, when contextual information is absorbed [41], such as when age becomes a role in sports performance or when an injury occurs, ego-related objectives are more “fragile” and might lead to the maladaptive pursuit of achievement. Hence, other contextually significant criteria for the success of biofeedback training and, indirectly, the effectiveness of strength training are relevant in addition to the level of alpha waves that the research team researched and analyzed.

## CONCLUSIONS

In conclusion, in this article, we explored the impact of differences in athletes’ motivation on their performance during bench press exercises, with a focus on the magnitude of external load and level of training, and how EEG biofeedback training can help optimize performance. One of the major variables influencing the efficiency of strength training, including bench press exercise, is the level of training. The more successfully an athlete uses motivation when exercising, the better their training, which translates into better results and a lower chance of injury. Yet, consistency in training as well as the right amount of time and effort put into it is required to reach a high level of training. When modifying the intensity of the exercise, one should take into account the level of training and, consequently, the planned objectives of boosting the efficacy of overcoming external loads during bench press workouts.

## Conflict of interest declaration

The authors decleared no conflict of interest.
